# Executive and Perceptual Distraction in Visual Working Memory

**DOI:** 10.1037/xhp0000413

**Published:** 2017-04-17

**Authors:** Richard J. Allen, Alan D. Baddeley, Graham J. Hitch

**Affiliations:** 1 School of Psychology, University of Leeds; 2 Department of Psychology, University of York

**Keywords:** attention, binding, distraction, visual working memory

## Abstract

The contents of visual working memory are likely to reflect the influence of both executive control resources and information present in the environment. We investigated whether executive attention is critical in the ability to exclude unwanted stimuli by introducing concurrent potentially distracting irrelevant items to a visual working memory paradigm, and manipulating executive load using simple or more demanding secondary verbal tasks. Across 7 experiments varying in presentation format, timing, stimulus set, and distractor number, we observed clear disruptive effects of executive load and visual distraction, but relatively minimal evidence supporting an interactive relationship between these factors. These findings are in line with recent evidence using delay-based interference, and suggest that different forms of attentional selection operate relatively independently in visual working memory.

## Public Significance Statement

This study demonstrates that the ability to encode and temporarily hold visual information depends both on how active (executive) attentional control is allocated, and whether there are (perceptual) distracting stimuli present in the visual environment. Across seven experiments, memory accuracy was always reduced when participants performed a more demanding task at the same time, and when to-be-remembered targets were accompanied by additional irrelevant items. These two forms of attentional interference appear to have relatively distinct impacts, indicating that we do not necessarily become more vulnerable to perceptual distraction when our executive attention is preoccupied by other tasks. This work provides new insights for the current debate on how working memory and attention might interact.

Attention and working memory are increasingly viewed as closely linked concepts, with a bidirectional relationship operating between where attention is allocated and what is temporarily retained. Research has demonstrated that the contents of working memory can guide attentional selection of stimuli in the external environment (e.g., Awh & Jonides, 2001; Downing, 2000; Hu, Xu, & Hitch, 2011; Olivers, Meijer, & Theeuwes, 2006; Soto, Heinke, Humphreys, & Blanco, 2005). Similarly, when mechanisms of attentional selection are directed toward stimuli, either through top-down control or automatic capture (e.g., Yantis, 2000), these stimuli are then more likely to be retained within working memory (e.g., Schmidt, Vogel, Woodman, & Luck, 2002; Vogel, Woodman, & Luck, 2005). In accordance with these findings, working memory may be conceptualized as the interface between internally oriented executive control and externally driven attention control (Chun, Golomb, & Turk-Browne, 2011; Yantis, 2000). Indeed, this interplay between working memory and attentional control may be closely related to general fluid intelligence (Shipstead, Harrison, & Engle, 2015; Unsworth & Spillers, 2010), thus illustrating the importance of this topic for our understanding of wider cognition. The current study focused on how different forms of attentional disruption (specifically, to-be-ignored environmental distraction and concurrent executive load) impact on working memory, and on the nature of the interaction between these factors.

Working memory resources appear to be important in managing the impacts of distraction on cognitive performance. This has been demonstrated in the work of Lavie and colleagues, using varying forms of concurrent load to examine perceptual and working memory determinants of selective attention (e.g., de Fockert, Rees, Frith, & Lavie, 2001; Konstantinou, Beal, King, & Lavie, 2014; Lavie, 1995, 2005, 2010). In a typical test of selective attention, participants attempt to make judgments to a target stimulus in the presence of a distractor stimulus that is congruent or incongruent with this target. In such circumstances, it is consistently found that incongruent distractors disrupt target judgments to a greater extent when the load on working memory imposed by an irrelevant concurrent task is increased (e.g., Konstantinou et al., 2014). These effects of working memory load, and their impacts on the ability to resist perceptual distractors, have been associated with increased prefrontal cortex activity, among other areas (de Fockert et al., 2001; McNab & Klingberg, 2008). In contrast, increasing perceptual load within the environment, for example by increasing the number of potential targets or their discriminability (Lavie, 1995), leads to reductions in the effects of a peripheral distractor in selective attention tasks. These patterns of findings have been claimed to illustrate the contrasting impacts of perceptual and working memory load on the ability to resist distraction (see Lavie, 2005, 2010, for reviews).

However, it remains an open question whether such conclusions based on findings from visual selective attention tasks generalize to visual working memory. With some exceptions, studies exploring visual working memory have tended to focus on memory for limited sets of information, studied in isolation. Participants are typically presented with visual stimuli and asked to try and encode all this information for the purposes of a subsequent test. However, this is not generally representative of the more cluttered and potentially distracting contexts in which visual working memory operates when information is encountered in the real-world. Normally, the individual is required to focus on information that is goal-relevant, and ignore or filter out distraction, with the success in doing so being an important determinant of performance (e.g., Zanto & Gazzaley, 2009). Studies exploring the effects of perceptual distraction on working memory have demonstrated that we are far from perfect in ignoring distraction, and that this ability relates to working memory capacity (Fukuda & Vogel, 2009; McNab & Dolan, 2014; Vogel, McCollough, & Machizawa, 2005). For example, Vogel et al. (2005) found that working memory capacity was closely related to an individual’s ‘filtering efficiency,’ that is, the ability to filter out irrelevant visual distractors and focus on targets (indexed by contralateral delay activity using EEG). Similarly, Fukuda and Vogel (2009; see also Gaspar, Christie, Prime, Jolicoeur, & McDonald, 2016) found individuals with superior working memory capacity (as measured by visual change detection performance) to be less susceptible to attentional capture from environmental distraction encountered within the target display. Fukuda and Vogel (2011) revised this interpretation in a later study, suggesting that attentional capture is fast and obligatory for all observers, but that individuals with lower working memory capacity take longer to recover from capture by distractors. Overall, these findings reflect mechanisms operating at the point of interaction between voluntary and involuntary modes of attention (Chun et al., 2011; Yantis, 2000). Fukuda and Vogel (2009) went on to suggest that such attentional control mechanisms play a central role in complex goal-directed behaviors such as abstract reasoning (Halford et al., 2007), thus aligning with the recent claims from Shipstead et al. (2015) of close links between working memory capacity, attentional control, and Gf.

One question that remains to be directly explored concerns the possible role that domain-general executive control resources might have in mediating interference caused by distractors encountered alongside to-be-remembered targets in visual working memory tasks. There is considerable evidence that central executive control is important in supporting visual working memory, as reflected in the highly reliable finding that concurrent performance of demanding verbal tasks (e.g., backward counting) during encoding and retention substantially reduces recognition and recall accuracy (e.g., Allen, Baddeley, & Hitch, 2006, 2014; Allen, Hitch, Mate, & Baddeley, 2012; Brown & Brockmole, 2010; Hu, Allen, Baddeley, & Hitch, 2016; Morey & Bieler, 2013; Morey & Cowan, 2004). Indeed, we have recently argued that executive control resources play an important role in maintaining representations in working memory over time when further to-be-remembered stimuli are encountered (Allen et al., 2014; Hu et al., 2016; Hu, Hitch, Baddeley, Zhang, & Allen, 2014).

More generally, control resources ascribed to the central executive will play a number of roles in visual working memory (and wider cognition). These include the kind of goal-directed, voluntary attentional control modes assumed to be operating in tasks requiring focus on targets and exclusion of distractors (e.g., Fukuda & Vogel, 2009; Lavie, 2005, 2010). Conceptions of working memory and attention would certainly suggest such a relationship. For example, in an earlier review of central executive function, Baddeley (1996) suggested that it may be important for focusing attention and ignoring distraction. However, despite such claims, and the growing body of research indicating links between distractor control and working memory capacity more generally (e.g., Fukuda & Vogel, 2009; Gaspar et al., 2016; McNab & Dolan, 2014), no previous study to our knowledge has examined how manipulating executive load might impact on the susceptibility of visual working memory to concurrently present visual distraction. We therefore attempted to address this in the present experimental work.

Using different stimulus sets and configurations, Experiments 1–3 examined the impact of distractors on memory for multiitem arrays of colors, shapes, and color-shape combinations, and explored whether this distractor effect increased in magnitude when participants performed a demanding verbal task concurrent with encoding and a brief retention interval. Experiment 4 then examined whether distractor effects increase as a function of the number of distractors present in the environment, and whether this mediates any relationship with executive control. These first four experiments were concerned with an executive load imposed prior to and during encoding, and a brief retention interval. Experiment 5 moved this load to a longer retention interval, to examine whether executive control is important in maintaining the distinction between targets and distractors over time. Finally, Experiments 6 and 7 examined concurrent distractor and executive load effects when targets were presented in a sequence, rather than in a simultaneous array (as in Experiments 1–5).

The contribution of central executive control resources was explored by having participants either perform articulatory suppression (repeating the same three-digit number across all trials) or backward counting in decrements of three from a random three-digit number presented at the start of each trial. This form of concurrent task manipulation has been used across a wide range of studies (e.g., Allen et al., 2006, 2009, 2012, 2014; Baddeley, Hitch, & Allen, 2009; Brown & Brockmole, 2010; Han & Kim, 2004; Karlsen, Allen, Baddeley, & Hitch, 2010; Yang, Gathercole, & Allen, 2014) and is effective in holding verbal activity approximately constant while varying executive load. In all cases, and regardless of the nature of the primary task, performance under conditions of backward counting is substantially less accurate than under articulatory suppression. When applying dual task logic, no task is likely to be pure, and we assume that concurrent backward counting will interfere with a range of cognitive processes. As with our previous use of this form of task, it is applied in the present experimental series with the intention of loading on domain-general attentional control resources, rather than attempting to target more specific mechanisms or abilities.

Within each experiment, we predicted a substantial disruptive effect of backward counting to emerge, in line with the assumption that executive control plays an important role in supporting visual working memory. We also predicted a negative impact of to-be-ignored visual distractors on target recall accuracy (consistent with Fukuda & Vogel, 2009; McNab & Dolan, 2014; Vogel et al., 2005). Finally, we tested the basic prediction that if the ability to focus on to-be-remembered targets and ignore simultaneously present distractors has a critical executive control component, the disruptive impact of distractors on target recall should substantially increase when performing a demanding concurrent task. Conversely, if these manipulations tap separable forms of attentional control that impinge somewhat independently on visual working memory, we should reliably observe two main effects but no interaction.

## Experiment 1

The first experiment was designed to establish whether to-be-ignored distractor stimuli encountered simultaneously with target items have an impact on immediate memory for colors, shapes, and colored shape combinations, and whether this is mediated by concurrent executive load. This was explored across stimulus conditions in this first experiment to establish whether effects of distractor and concurrent task are consistent across type and complexity of to-be-remembered stimulus. In addition, recall of color + shape combinations relative to the individual color and shape conditions provides a method of examining memory for bound object representations (see Gajewski & Brockmole, 2006), and therefore a means of examining whether feature bindings are relatively more vulnerable to environmental distraction (though we had no a priori predictions on this point). Memory was tested by spatially cued recall, a method that has been shown to be sensitive to disruption from a different form of environmental interference, namely a visual distractor suffix presented after target offset (Allen, Castella, Ueno, Hitch, & Baddeley, 2015).

### Method

#### Participants

Thirty-six undergraduate students (25 females) aged between 18 and 34 years took part in the 45-min experiment, for financial payment or course credit. The study was approved by the research ethics committee at the Department of Psychology, University of York.

#### Materials

Testing was controlled on a Macintosh laptop with a 15-in screen, using a SuperCard program. All stimuli were simple shapes measuring 70 × 70 mm (viewed at a distance of approximately 50 cm, thus subtending a visual angle of 0.8°), presented on a white background. A pool of eight shapes (circle, chevron, triangle, star, arch, cross, diamond, flag) and eight colors (black, red, blue, green, yellow, gray, turquoise, purple) were used in this experiment. Shapes were presented as unfilled three-point black outlines in the shape condition, while colors were presented as identical neutral formless shapes (“blobs”) in the color condition (e.g., Allen et al., 2014). Combination condition stimuli were three-point black outlines containing different color in-fills.

#### Design and procedure

This experiment followed a 3 × 2 × 2 repeated measures design, with stimulus condition (color; shape; color + shape), distractor (no-distractors; distractors), and concurrent task (articulatory suppression; backward counting) as factors. Stimulus condition and concurrent task were blocked, while the presence or absence of distractors was manipulated within each stimulus and task block as randomly intermixed trials. Each condition started with six practice trials, three of which were ‘no distractor’ trials while three were ‘distractor’ trials. This was followed by 36 test trials (18 no distractor and 18 distractor trials). Condition order was counterbalanced across participants.

At the start of the session, the set of eight shapes and eight colors were displayed on screen, along with the names associated with each, to ensure that participants knew which verbal label applied to which feature (for the purposes of the verbal response format). The first block of practice and test trials then followed.

**Figure 1 fig1:**
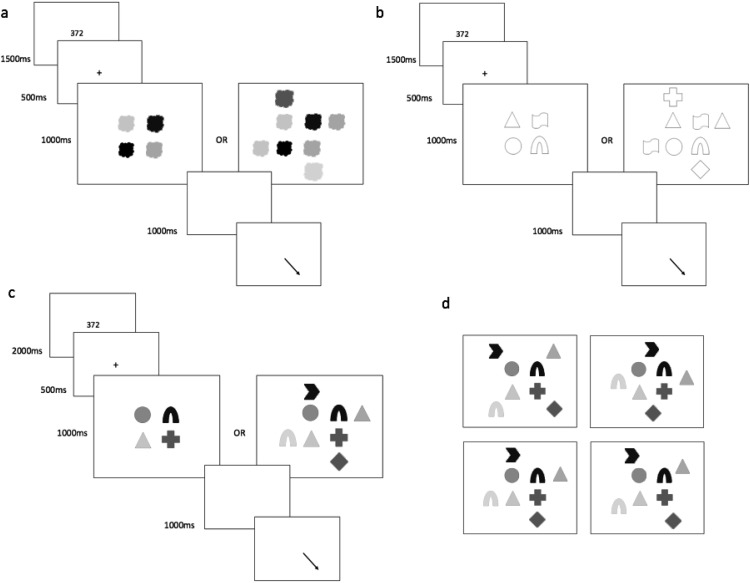
Schematic illustration of trial procedure in Experiment 1, for no-distractor and distractor trials in the (a) color condition, (b) shape condition, and (c) color + shape condition. As illustrated in (d), using the color + shape condition as an example, the four targets were always presented at screen center, and the four distractors in one of four surrounding configurations. Sizes are not to scale, and shades of gray represent different colors.

The presentation sequence in each trial is illustrated in Figure 1. Each trial started with the presentation of a three-digit number on screen for 1,500 ms. This number was always *123* in the articulatory suppression condition, with participants required to repeatedly articulate this (i.e., “one hundred twenty-three, one hundred twenty-three” from fixation cross to recall cue, at a steady rate. In the backward counting condition, a different three-digit number (e.g., *355*) was randomly generated and presented on screen in each trial. Participants attempted to count backward in decrements of three from this number (e.g., “three hundred fifty-five, three-hundred fifty-two, three hundred forty-nine, . . . etc.), up to the point of the recall cue.[Fig-anchor fig1]

Following a 1,500-ms blank screen, each trial commenced with a fixation cross at screen center for 500 ms, followed by a 250-ms blank screen delay. The four target stimuli were then simultaneously presented around screen center for 1,000 ms, within an invisible 2 × 2 grid (with a separation of approximately 1° visual angle between each item). Target shape and/or color were selected at random within each trial, with no repetition between targets. In ‘distractor’ trials, the target stimuli were surrounded by four additional to-be-ignored stimuli, drawn from the same category (i.e., color, shape, or color-shape combinations) as the target stimulus condition, again without feature repetition. Feature overlap between target and distractor features was permitted and unconstrained. The location of the distractors randomly varied between four possible configurations surrounding the central targets (see Figure 1d). In all cases they were evenly distributed and set approximately 2° visual angle apart from each other, and approximately 1° from the nearest target stimulus.

The target (and distractor) display was followed by a blank screen retention interval of 1,000 ms. The recall cue was then displayed. This consisted of a black arrow pointing toward a corner of the screen. This arrow cued the relative location of one of the target items within the 2 × 2 grid (e.g., top left), with participants required to recall out loud the item occupying that position. Participants were encouraged to guess or respond with ‘do not know’ if they felt they did not know the answer. The experimenter manually recorded responses from the visual working memory and backward counting tasks.

### Results and Discussion

Data in this and all subsequent experiments were analyzed using ANOVA and appropriate follow-up comparisons (corrected using Bonferroni-Holm). Bayesian ANOVA (JASP Team, 2016) were also performed, to identify the model with the strongest support. In each case, the relative support for the key interaction between distraction and concurrent task is also noted.

**Figure 2 fig2:**
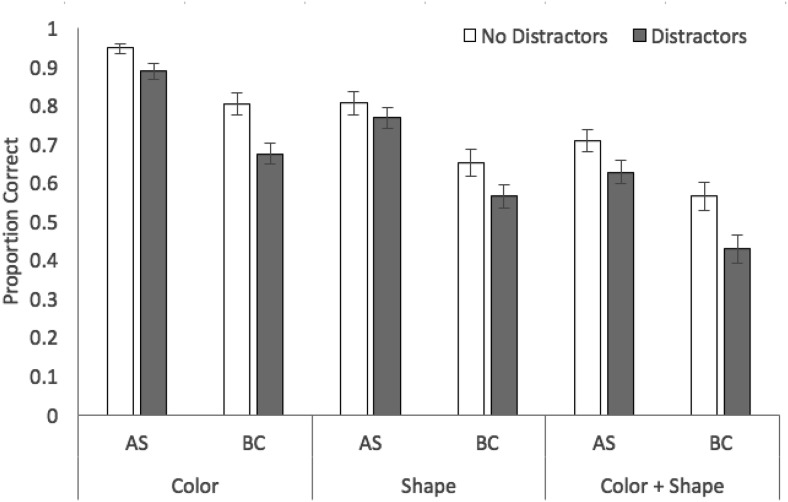
Proportion correct (with standard error in error bars) in Experiment 1 as a function of stimulus condition, distractors, and concurrent task.

Proportion correct in probed recall of target stimuli in each stimulus, concurrent task, and distractor condition is displayed in Figure 2. A 3 × 2 × 2 analysis of variance revealed significant effects of stimulus condition, *F*(2, 70) = 111.15, *MSE* = .02, *p* < .001, η_*p*_^2^ = .76, with recall accuracy higher for color (mean .83, *SE* .02) than shape (.70, .02) or color + shape (.58, .03), and the latter two conditions also differing (*p* < .001 in all cases, Bonferroni-Holm corrected). These relative recall performance levels in each stimulus condition closely mirror those observed previously using recognition (e.g., Allen et al., 2006). There were also significant effects of concurrent task, *F*(1, 35) = 101.60, *MSE* = .03, *p* < .001, η_*p*_^2^ = .74, with performance during articulatory suppression superior to the backward counting condition, and distractors, *F*(1, 35) = 71.94, *MSE* = .01, *p* < .001, η_*p*_^2^ = .67, with a negative effect of distractor presence on target memory accuracy. There was a significant interaction between concurrent task and distractors, *F*(1, 35) = 7.86, *MSE* = .01, *p* = .008, η_*p*_^2^ = .18, with a larger impact of distractors under backward counting, *t*(35) = 6.69, *p* < .001, *d* = .68, than under articulatory suppression, *t*(35) = 5.42, *p* < .001, *d* = .48. The interactions between stimulus condition and distractors, *F*(2, 70) = 2.52, *MSE* = .01, *p* = .09, η_*p*_^2^ = .07, and stimulus condition and concurrent task, *F*(2, 70) = .05, *MSE* = .01, *p* = .96, η_*p*_^2^ = .01, were not significant, nor was the three-way interaction *F*(2, 70) = .11, *MSE* = .01, *p* = .90, η_*p*_^2^ = .01. Bayesian ANOVA indicated strongest support for the model containing the three main effects (concurrent task, stimulus condition, distraction) plus the interaction between task and distraction (BF >1,000 vs. the null-only model), though this was only 2.3:1 more likely than the main effects-only model.[Fig-anchor fig2]

Although the above analyses indicate some equivocal support for the presence of an interaction between concurrent task and distraction, this may partly reflect scaling issues within the data in this experiment. To test this, data were also analyzed following arcsine square root transformation.^1^ Although this still produced a significant task by distraction interaction, *F*(1, 35) = 4.74, *MSE* = .01, *p* = .042, η_*p*_^2^ = .11, the difference in distractor effect sizes between articulatory suppression (*Cohen’s d* = .53) and backward counting (*d* = .69) was not large. Furthermore, a Bayesian ANOVA on the transformed data indicated evidence favoring the model containing the main effects only (BF >1,000 vs. the null-only model), over the model with main effects + the task by distraction interaction, though only by a factor of 1.4:1.

Although our primary focus was on the visual task, we also analyzed backward counting performance. Mean number of correct counting responses per trial are displayed in Table 1 (for all experiments). A 3 × 2 repeated measures ANOVA on the Experiment 1 data revealed significant effects of stimulus condition, *F*(2, 70) = 3.49, *MSE* = .18, *p* = .036, η_*p*_^2^ = .09, distractors, *F*(1, 35) = 9.16, *MSE* = .15, *p* = .005, η_*p*_^2^ = .21, but no interaction, *F*(2, 70) = .31, *MSE* = .01, *p* = .74, η_*p*_^2^ = .01. A Bayesian ANOVA indicated that the best model contained stimulus condition and distractors (*BF* = 14 vs. the null), but this was only 1.6 times more likely than the model excluding distractors and only containing stimulus condition. Thus, counting performance outcomes resemble those obtained in the primary visual task, with a significant effect of distraction but only weak or anecdotal evidence for this effect according to Bayesian analysis.[Table-anchor tbl1]

The large deleterious effect of backward counting on visual working memory signals an important contribution from central executive control resources to this task (e.g., Allen et al., 2006, 2014). The consistent effect of visual distraction, with target recall accuracy reduced when to-be-ignored distractors were also present in the environment, is in line with previously observed findings in suggesting that participants are not always able to filter out irrelevant visual stimuli and focus purely on target encoding and retention (e.g., Fukuda & Vogel, 2009; McNab & Dolan, 2014; Vogel et al., 2005). These load and distractor effects did not interact with stimulus condition, emerging consistently across conditions measuring memory for color, shape, and colored shape combinations. Although a statistically significant interaction was observed between concurrent task and distraction in the proportion correct data, and an effect of distraction was also found on backward counting performance, the Bayes Factors supporting each of these outcomes fall into the ‘weak’ or ‘anecdotal’ range (Jarosz & Wiley, 2014). Furthermore, for the primary visual task, the Bayes Factor slightly favored the absence of this effect after data were transformed to address potential scaling issues.

## Experiment 2

The first experiment produced clear impacts of executive load and distraction on visual WM. Although some evidence was observed for an interaction between load and distraction, the support for this (as indicated by Bayesian analysis) was weak. Given these equivocal outcomes, it is important to further explore whether a relationship between load and distraction can be reliably observed across different experimental contexts. Experiment 2 sought to explore this by extending the methodology from Experiment 1 to a different stimulus set, specifically, photographic images of real objects. No previous studies to date have examined how concurrent distractors impinge on visual working memory when stimuli constitute familiar and meaningful items.

### Method

#### Participants

Twenty-four undergraduate students (15 females) aged between 18 and 29 years took part, for financial payment or course credit. The study was approved by the research ethics committee at the Department of Psychology, University of York.

#### Materials

**Figure 3 fig3:**
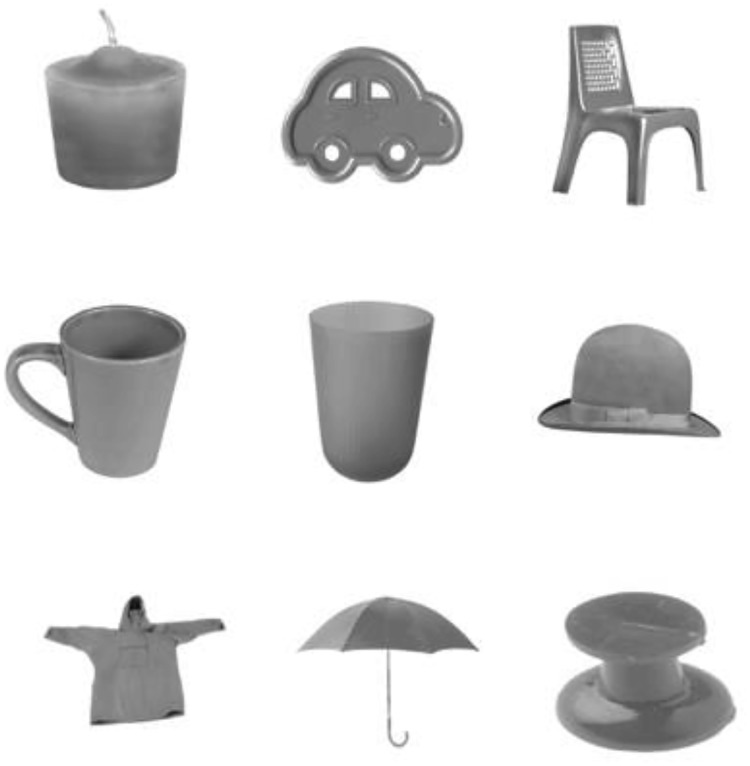
Object stimuli used in Experiment 2, showing (left to right and top to bottom), candle, car, chair, cup, glass, hat, jacket, umbrella, plus the neutral item (tack) used in the color condition.

All stimuli measured 60 × 60 mm (viewed at a distance of approximately 50 cm, thus subtending a visual angle of 0.69°), presented on a white background. A pool of objects was selected, with the constraint that such objects do not have a prototypical color. Photographic versions of these objects were then developed, in each of the experimental colors. In the shape condition, shapes were drawn from a pool of 8 (candle, car, chair, cup, glass, hat, jacket, umbrella) and presented in a neutral color (brown). For the color condition, stimuli were drawn from a pool of 8 colors (blue, green, gray, orange, purple, red, turquoise, yellow) and presented in the form of a neutral shape (tack). Color + shape stimuli constituted colored shape versions of each of these items. The object stimulus set is displayed in Figure 3.[Fig-anchor fig3]

#### Design and procedure

Aside from the use of an alternative stimulus set, methodology was identical to that used in Experiment 1.

### Results and Discussion

**Figure 4 fig4:**
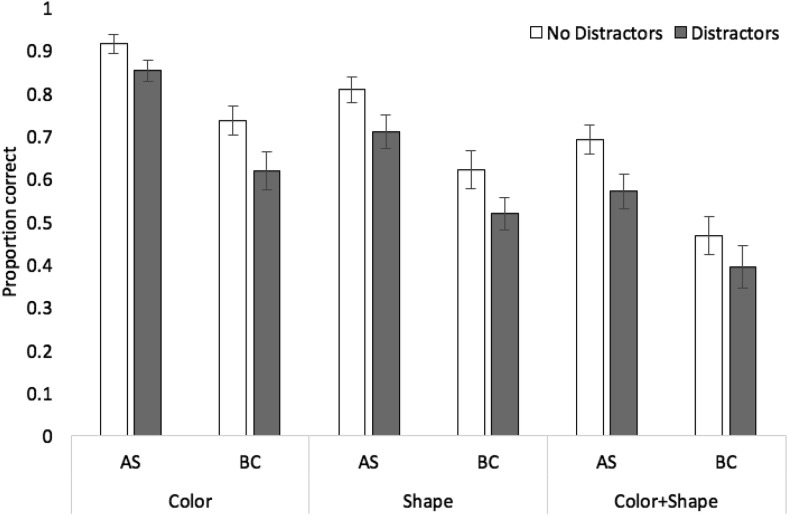
Proportion correct (with standard error in error bars) in Experiment 2 as a function of stimulus condition, distractors, and concurrent task.

Proportion correct in probed recall of target stimuli in each stimulus, concurrent task, and distractor condition is displayed in Figure 4.[Fig-anchor fig4]

A 3 × 2 × 2 analysis of variance revealed significant effects of stimulus condition, *F*(2, 46) = 74.27, *MSE* = .02, *p* < .001, η_*p*_^2^ = .76, with recall accuracy higher for color (mean .78, *SE* .03) than shape (.66, .04) or color + shape (.53, .04), and the latter two conditions also differing (*p* < .001 in all cases, Bonferroni-Holm corrected). There were also significant effects of concurrent task, *F*(1, 23) = 68.60, *MSE* = .04, *p* < .001, η_*p*_^2^ = .75, and distractors, *F*(1, 23) = 32.32, *MSE* = .02, *p* < .001, η_*p*_^2^ = .58. The interaction between concurrent task and distractors was not significant, *F*(1, 23) = .02, *MSE* = .01, *p* = .892, η_*p*_^2^ = .001. Similarly, the interactions between stimulus condition and distractors, *F*(2, 46) = .07, *MSE* = .01, *p* = .94, η_*p*_^2^ = .003, stimulus condition and concurrent task, *F*(2, 46) = .15, *MSE* = .01, *p* = .86, η_*p*_^2^ = .01, were not significant, nor was the three-way interaction *F*(2, 46) = 2.01, *MSE* = .01, *p* = .15, η_*p*_^2^ = .08. Bayesian ANOVA indicated strongest support for the model containing the main effects only (BF >1,000 vs. the null-only model), preferring this over the model with main effects plus the distractor by task interaction by a factor of 5.8:1.

Backward counting performance (see Table 1) was examined in a 3 × 2 repeated measures ANOVA, which revealed a marginal effect of stimulus condition, *F*(2, 46) = 3.18, *MSE* = .07, *p* = .051, η_*p*_^2^ = .12, but no effect of distractors, *F*(1, 23) = .01, *MSE* = .01, *p* = .93, η_*p*_^2^ = .00, or the interaction, *F*(2, 46) = .44, *MSE* = .02, *p* = .65, η_*p*_^2^ = .02. A Bayesian ANOVA indicated strongest support for the model containing stimulus condition only (*BF* = 11 vs. the null-only model), preferring this over the model also containing distractors by a factor of 5:1.

This experiment therefore replicated each of the main effects from Experiment 1 using a meaningful stimulus set consisting of familiar manmade objects. Furthermore, the absence of an interaction between executive load and visual distraction builds on outcomes from the first experiment concerning the absence of clear evidence supporting a critical role for the executive control in minimizing impacts of environmental interference on visual WM.

## Experiment 3

In the preceding experiments, distractor stimuli have always been presented in locations surrounding the four central target items. This spatial separation and the predictability of its configuration may reduce the difficulty of distractor exclusion, possibly enabling an easier and more automatic ‘zooming in’ process of selective attention to the central targets. In Experiment 3, we examined whether executive control becomes more important for distractor control when to-be-remembered and to-be-ignored stimuli are randomly intermixed. To distinguish the two classes of stimuli, each target was surrounded by a black outline square during initial presentation of the display. This procedure produces configurations in which each target location is unpredictable, is not distinct from distractor item’s locations, and indeed is often noncontiguous with the locations of the other target items. Previous work has indicated that participants can recall items from noncontiguous locations, though the ability to do so at least partly reflects suppression of interference from unattended stimuli (Awh & Jonides, 2001). Experiment 4 therefore examined whether this is critically dependent on availability of executive control resources.

### Method

#### Participants

Twenty-four undergraduate students (19 females) aged between 18 and 32 years took part, for financial payment or course credit. The study was approved by the research ethics committee at the Department of Psychology, University of York.

#### Materials, design, and procedure

Overall design of this experiment was based closely on Experiments 1 and 2, manipulating concurrent task (articulatory suppression vs. backward counting), stimulus condition (color, shape, color + shape), and distraction (no distractors vs. 4 distractors). Each condition started with four practice trials (two no-distractor and two distractor trials). This was followed by 32 test trials (16 no-distractor and 16 distractor trials). Condition order was counterbalanced across participants.

**Figure 5 fig5:**
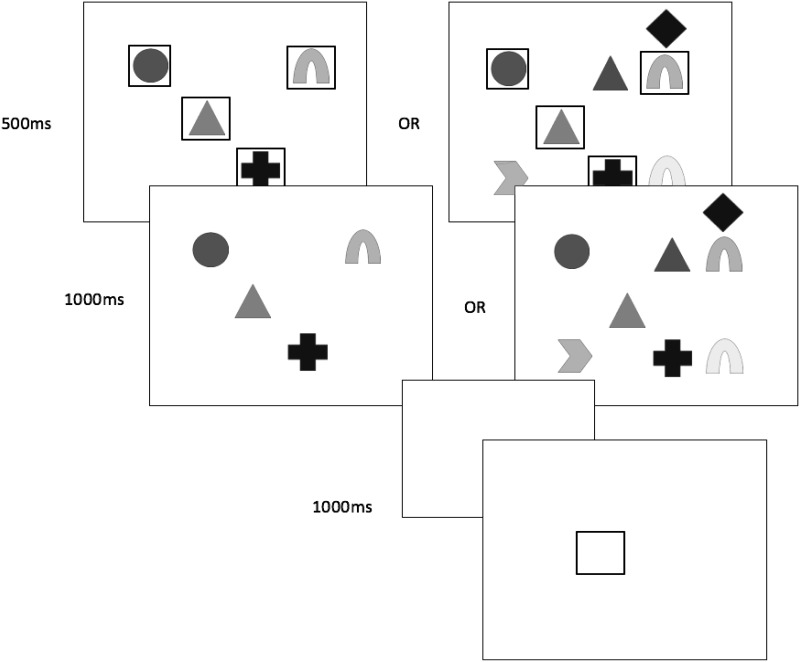
Schematic illustration of trial procedure in Experiment 3, for no-distractor (left display) and distractor (right display) trials using the color + shape condition as an illustrative example. Sizes are not to scale, and shades of gray represent different colors.

All target and distractor stimuli were taken from Experiment 1. The presentation sequence in each trial is illustrated in Figure 5. In this experiment, the four target stimuli were simultaneously presented in randomly selected locations within an invisible 4 × 4 grid (with a separation of approximately .70° visual angle between each item). On distractor-present trials, four distractor stimuli were also presented within this same grid. For all trials, stimulus exposure duration was 1,500 ms, with target items surrounded by a black square outline (1.4° in size) for the first 500 ms of presentation. These target cues were removed for the final 1,000 ms of presentation, meaning that targets and distractors were visually and spatially undifferentiated for this brief period. At the recall phase, a single black square outline was presented at one of the four target locations, with participants required to recall out loud the item occupying that position.[Fig-anchor fig5]

### Results and Discussion

**Figure 6 fig6:**
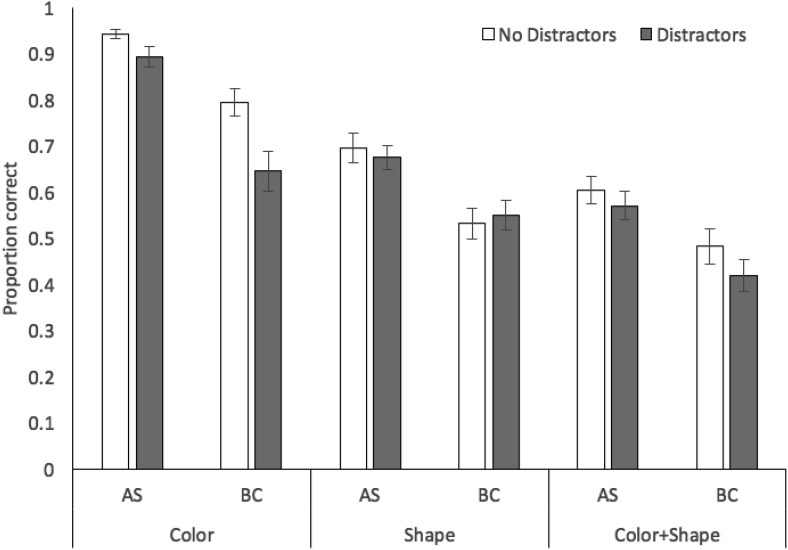
Proportion correct (with standard error in error bars) in Experiment 3 as a function of stimulus condition, distractors, and concurrent task.

Proportion correct in each condition is displayed in Figure 6. A 3 × 2 × 2 analysis of variance revealed significant effects of stimulus condition, *F*(2, 46) = 120.24, *MSE* = .02, *p* < .001, η_*p*_^2^ = .84, with recall accuracy higher for color (mean .82, *SE* .03) than shape (.61, .03) or color + shape (.52, .03), and the latter two conditions also differing (*p* < .001 in all cases, Bonferroni-Holm corrected). There were also significant effects of concurrent task, *F*(1, 23) = 102.73, *MSE* = .04, *p* < .001, η_*p*_^2^ = .82, and distractors, *F*(1, 23) = 12.35, *MSE* = .01, *p* = .002, η_*p*_^2^ = .35. The key interaction between concurrent task and distractors was not significant, *F*(1, 23) = 1.26, *MSE* = .01, *p* = .274, η_*p*_^2^ = .05, nor was the task by stimulus condition, *F*(2, 46) = 1.97, *MSE* = .01, *p* = .15, η_*p*_^2^ = .08. There was an interaction between stimulus condition and distractors, *F*(2, 46) = 3.31, *MSE* = .02, *p* = .045, η_*p*_^2^ = .13, and a three-way interaction *F*(2, 46) = 3.70, *MSE* = .01, *p* = .032, η_*p*_^2^ = .14. These latter interactions can be attributed to a somewhat larger concurrent task effect for the color condition when distractors were present, though this is likely to simply reflect the very high performance levels under AS/no-distractors in this condition. Indeed, when the data were transformed using arcsine square root to minimize scaling issues, this three-way interaction was no longer significant, *F*(2, 46) = 2.67, *MSE* = .01, *p* = .08, η_*p*_^2^ = .10 (all other outcomes remained the same). A Bayesian ANOVA (on the untransformed data) indicated evidence favoring the model containing the main effects plus the stimulus condition by distractor interaction (BF >1,000 vs. the null-only model), though this was only preferred over the main effects-only model by 1.8:1. In terms of evidence for the key interaction between concurrent task and distraction, the Bayesian ANOVA preferred the main effects-only model (i.e., without the interaction) by a factor of 3.6 to 1.[Fig-anchor fig6]

Backward counting performance (see Table 1) was also examined, though note that data from 9 participants was missing due to experimenter error, so this analysis was only carried out on 15/24 participants. A 3 × 2 repeated measures ANOVA revealed no effects of stimulus condition, *F*(2, 28) = 1.03, *MSE* = .06, *p* = .37, η_*p*_^2^ = .07, distractors, *F*(1, 14) = .52, *MSE* = .04, *p* = .48, η_*p*_^2^ = .04, or the interaction, *F*(2, 28) = .81, *MSE* = .6, *p* = .45, η_*p*_^2^ = .06. A Bayesian ANOVA indicated strongest support for the null model, preferring this over the model also containing distractors by a factor of 4:1.

Thus, intermixing the targets and distractors in unpredictable configurations did not produce any evidence supporting a role for executive control in distractor exclusion, with a nonsignificant interaction and Bayes Factor supporting the main effects-only model.

## Experiment 4

Using different stimulus sets and display configurations, the first three experiments provide clear evidence for effects of executive load and distraction, but minimal support for an interactive relationship between these factors. Experiment 4 was designed to further explore this issue, examining whether the number of distractors present in the environment is an important factor in the magnitude of their disruptive effects, and the relationship with attentional control. So far, we have examined memory for four targets, in the presence or absence of four additional distractor items. In Experiment 4, we examined recall performance under conditions of zero, one, four, or eight distractors. It may be that distractor interference effects increase with the number of distractors present, and that the role of executive control resources correspondingly become more critical in each case. As distractor and load effects did not vary with stimulus condition in Experiments 1–3, we simplified the design in Experiment 4 by limiting exploration to the color + shape stimulus condition.

### Method

#### Participants

Thirty-four participants (30 females) aged 18–35 years took part in this experiment, for course credit or payment. The study was approved by the research ethics committee at the School of Psychology, University of Leeds.

#### Materials

The materials from Experiment 1 were used again in this experiment.

#### Design and procedure

This experiment followed a 4 × 2 repeated measures design, with distractor level (no-distractors; 1 distractor; 4 distractors; 8 distractors) and concurrent task (articulatory suppression; backward counting) as factors. Concurrent task was blocked and order counterbalanced between participants, whereas distractors were manipulated within each task block as randomly intermixed trials. Each condition started with four practice trials, with one drawn from each of the four distractor trial types. This was followed by 80 test trials, constituting 20 no-distractor trials and 20 trials each involving 1, 4, or 8 distractors.

**Figure 7 fig7:**
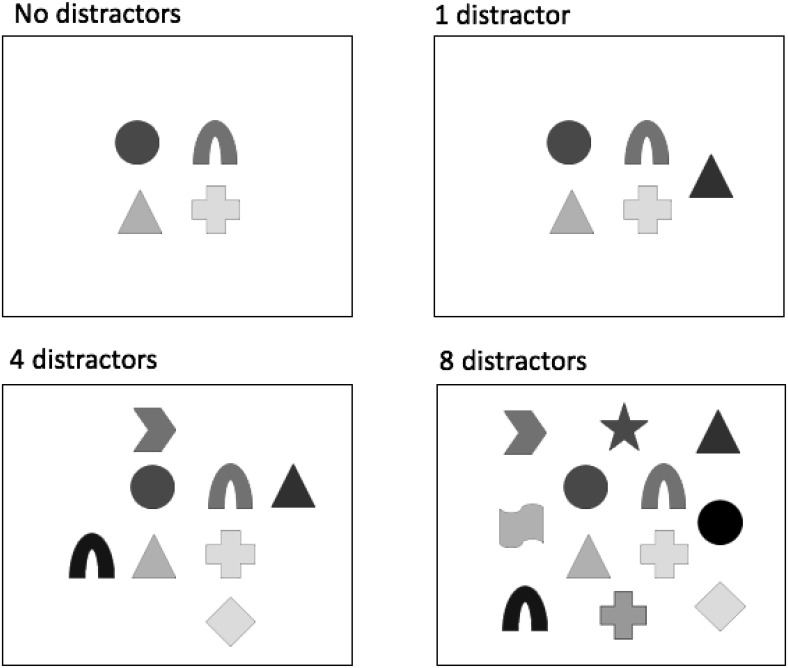
Schematic illustration (not to scale) of display configurations from each of the distractor conditions in Experiment 4.

Trial details and concurrent task implementation followed the same procedure as Experiments 1 and 2. Examples of possible distractor configurations are displayed in Figure 7. The same set of possible distractor locations was used for all conditions. Within the 1-distractor trials, a single colored shape was presented in one of these 16 locations surrounding the target set. For 4-distractor trials, as in Experiment 1, these stimuli appeared in one of four (randomly selected) configurations, evenly distributed around the targets. In the case of 8-distractor trials, the distractors appeared in one of two randomly selected configurations, again evenly distributed around the targets. Feature overlap between targets and distractors was controlled so that each distractor stimulus contained one feature (either a color or shape) that appeared within the target set, whereas the remaining feature was drawn from the wider experimental set.[Fig-anchor fig7]

### Results and Discussion

**Figure 8 fig8:**
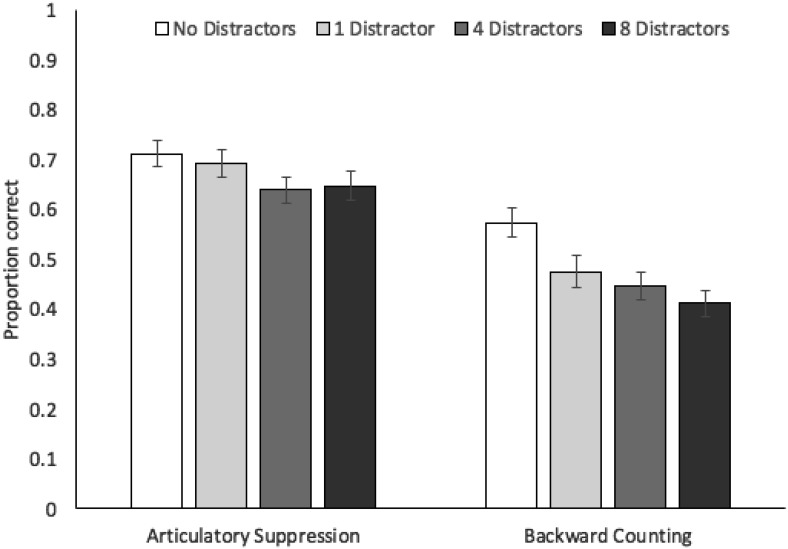
Proportion correct (with standard error in error bars) in Experiment 4 as a function of distractors and concurrent task.

Proportion correct in probed recall of target stimuli in each concurrent task and distractor condition is displayed in Figure 8. A 2 × 4 repeated measures ANOVA produced significant effects of concurrent task, *F*(1, 33) = 100.78, *MSE* = .03, *p* < .001, η_*p*_^2^ = .75, and distractors, *F*(3, 99) = 17.35, *MSE* = .01, *p* < .001, η_*p*_^2^ = .35. Further comparisons (Bonferroni-Holm corrected), collapsing across concurrent task conditions, indicated that accuracy on 0-distractor trials (mean .64, *SE* .02) was higher than on trials with 1 distractor (mean .58, *SE* .03, *p* = .002), 4 distractors (mean .54, *SE* .02, *p* < .001), and 8 distractors (mean .53, *SE* .03, *p* < .001). Accuracy was also higher on trials with 1 distractor, relative to 4 distractors (although this was marginally nonsignificant after correction, *p* = .054), or 8 distractors (*p* = .004), whereas 4- and 8-distractor conditions did not differ (*p* = .42). There was also a significant interaction between concurrent task and distractors, *F*(3, 99) = 3.79, *MSE* = .01, *p* = .014, η_*p*_^2^ = .10. However, Bayesian ANOVA failed to distinguish between models, and slightly preferred the main effects-only model versus the model also containing the interaction, by a factor of 1.4:1 (BF > 1,000 vs. the null-only model).[Fig-anchor fig8]

We also examined the effects of distraction and concurrent task purely for trials in which distractors were always present, but varied in number (i.e., focusing on 1-, 4-, and 8- distractor trials). A 2 × 3 repeated measures ANOVA produced significant effects of concurrent task, *F*(1, 33) = 106.31, *MSE* = .02, *p* < .001, η_*p*_^2^ = .76, and distractors, *F*(2, 66) = 5.79, *MSE* = .01, *p* = .005, η_*p*_^2^ = .15, but no interaction, *F*(2, 66) = 1.08, *MSE* = .01, *p* = .35, η_*p*_^2^ = .03. Bayesian analysis preferred the main effects-only model (BF > 1,000 vs. the null), with a Bayes Factor of 6.2 to 1 against inclusion of the interaction.

Backward counting performance (see Table 1) was examined in a repeated measures ANOVA, which revealed no effects of number of distractors, *F*(3, 99) .37, *MSE* = .02, *p* = .78, η_*p*_^2^ = .01. A Bayesian ANOVA indicated strongest support for the null model, preferring this over the model also containing distractors by a factor of 17:1.

This experiment therefore replicated the negative effects of executive load and distraction that were observed in Experiments 1–3. In the latter case, we observed some evidence of increasing distractor interference when more were present in the environment. The presence of four or eight distractors alongside the four to-be-remembered targets led to reduced recall accuracy, relative to a single distractor, suggesting that more distraction can lead to more interference. However, recall accuracy was equivalent in four- and eight-distractor trials, indicating a certain plateauing of this effect. One possibility is that the likelihood of distractors being processed depends in part on the availability of perceptual processing resources, with these resources perhaps being exceeded before we get to eight distractors. Indeed, within Lavie’s approach (Lavie, 1995, 2005, 2010), the processing (and therefore, the influence) of additional distractor items is minimized once perceptual processing capacity is overloaded. This approach has been developed to capture outcomes from selective attention studies showing reduced disruption caused by distractors when the perceptual load associated with target processing is high (e.g., Lavie et al., 2004). Within the current experiment, in contrast, perceptual load varies with manipulation of distractor number. Lavie’s approach might account for the present findings by assuming that target memory accuracy reduces as more distractors are encountered, up to the point at which perceptual load exceeds processing capacity. Once these limits are reached, any further distractor items that are present will not receive sufficient perceptual processing for them to disrupt targets.

There was also an interaction between concurrent task and distraction, with examination of performance in each condition indicating somewhat reduced accuracy on distractor-present trials under backward counting, relative to no-distractor trials. However, Bayes Factor support for this interaction was entirely equivocal, with evidence slightly favoring the absence of this interaction within the model. Furthermore, when comparing trials featuring 1, 4, or 8 distractors, we observed evidence against the interaction with concurrent task. Similarly, there were no differences in counting rates between the stimulus conditions. These findings would run counter to the assumption that each to-be-ignored stimulus requires a certain degree of executive control to exclude or gate it out of working memory; such an assumption would predict increasing concurrent attentional load effects the more distractors are present. Thus, even when increasing the number of distractors that are present, we do not see convincing evidence for a critical role of executive resources in minimizing interference.

## Experiment 5

All experiments so far have focused on how executive resources during encoding and a brief (1-s) delay might contribute to target encoding and mediation of interference caused by simultaneously present distraction. A related question is whether executive control is important in maintaining the distinction between targets and distractors during a longer retention interval. Experiment 5 therefore shifted the executive load manipulation to a 5-s delay between target offset and test. Previous research has demonstrated that domain-general attention is required for visual WM maintenance (Morey & Bieler, 2013; Morey & Cowan, 2005), thus predicting a concurrent task effect to again emerge in this experiment. Our interest lay in whether to-be-ignored distractors would particularly interfere with target memory during a longer retention interval when executive control resources were directed to a more demanding concurrent task.

### Method

#### Participants

Twenty-four undergraduate students (13 females) aged between 18 and 30 years took part, for financial payment or course credit. The study was approved by the research ethics committee at the Department of Psychology, University of York.

#### Materials, Design, and Procedure

Design, materials, and procedure were closely based on Experiment 1. Each condition started with 4 practice trials, followed by 20 test trials (10 no-distractor and 10 distractor). The key differences in procedure were implemented at the retention phase, with a blank screen delay of 5,000 ms inserted in all trials. On AS trials, participants continued to repeat “*123*” from display onset up to the point of the recall cue (see Experiment 1). On BC trials, participants performed AS during encoding only. Immediately following display offset, a three-digit number was aurally presented through speakers, with participants required to count backward in 3s from this number, up to test cue presentation.

### Results and Discussion

**Figure 9 fig9:**
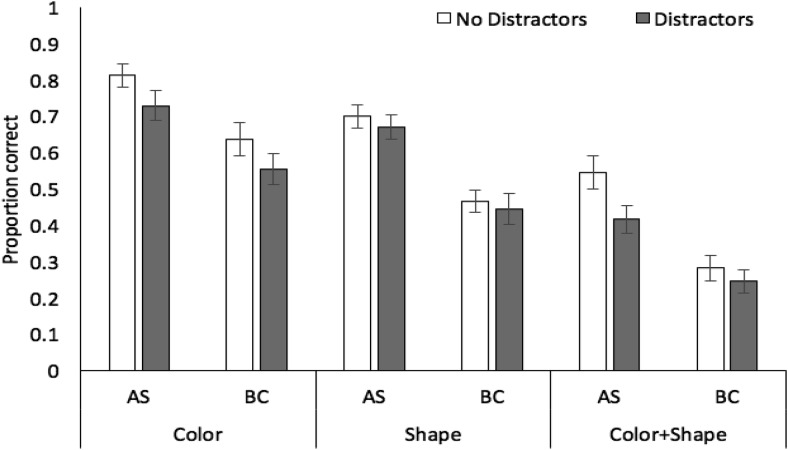
Proportion correct (with standard error in error bars) in Experiment 5 as a function of stimulus condition, distractors, and concurrent task.

Proportion correct in each condition is displayed in Figure 9. A 3 × 2 × 2 analysis of variance revealed significant effects of stimulus condition, *F*(2, 46) = 75.58, *MSE* = .03, *p* < .001, η_*p*_^2^ = .77, with recall accuracy higher for color (mean .68, *SE* .03) than shape (.57, .03) or color + shape (.37, .03), and the latter two conditions also differing (*p* < .001 in all cases, Bonferroni-Holm corrected). There were also significant effects of concurrent task, *F*(1, 23) = 58.06, *MSE* = .05, *p* < .001, η_*p*_^2^ = .72, and distractors, *F*(1, 23) = 20.62, *MSE* = .01, *p* < .001, η_*p*_^2^ = .47. The interaction between concurrent task and distractors was not significant, *F*(1, 23) = 1.03, *MSE* = .02, *p* = .322, η_*p*_^2^ = .004. Similarly, the interactions between stimulus condition and distractors, *F*(2, 46) = 1.64, *MSE* = .02, *p* = .21, η_*p*_^2^ = .07, stimulus condition and concurrent task, *F*(2, 46) = .76, *MSE* = .03, *p* = .48, η_*p*_^2^ = .03, were not significant, nor was the three-way interaction *F*(2, 46) = 1.39, *MSE* = .01, *p* = .26, η_*p*_^2^ = .06. Bayesian ANOVA indicated strongest support for the model containing just the main effects (BF >1,000, vs. the null-only model), preferring this over the model with main effects plus the distractor by task interaction by a factor of 4.2:1.[Fig-anchor fig9]

Backward counting performance (see Table 1) was also examined. A 3 × 2 repeated measures ANOVA revealed no effects of stimulus condition, *F*(2, 46) = 2.38, *MSE* = .09, *p* = .10, η_*p*_^2^ = .09, distractors, *F*(1, 23) = 3.75, *MSE* = .3, *p* = .065, η_*p*_^2^ = .14, or the interaction, *F*(2, 46) = 1.46, *MSE* = .03, *p* = .24, η_*p*_^2^ = .06. A Bayesian ANOVA indicated strongest support for the null model, preferring this over the model also containing distractors by a factor of 2:1. It should be noted that counting scores were numerically slightly higher on trials containing distractors, relative to target-only trials.

The delay-based implementation of concurrent load in this experiment indicates that executive control continues to be important in visual WM throughout retention (Morey & Bieler, 2013; Morey & Cowan, 2005). However, this is not a crucial factor in maintaining the distinction between targets and distractors, with no interaction observed between these factors.

## Experiment 6

The final two experiments in this series examined how executive control and distraction impact on visual WM when targets are presented serially, rather than in a single simultaneous array. We have previously demonstrated a particular profile of performance across short sequences of visual stimuli, with early sequence items less accurately recalled and requiring attentional support to for their maintenance, in contrast to a recency advantage at the final item that emerges even under executive load but is relatively more vulnerable to a postsequence distractor ‘suffix’ (Allen et al., 2014; Hu et al., 2014, 2016). Memory for feature combinations also seems to be more vulnerable during serial presentation, particularly at early positions (Allen et al., 2006, 2014; Brown & Brockmole, 2010; Brown, Niven, Logie, Rhodes, & Allen, 2017). However, it remains to be seen how concurrent distraction impinges on serial visual WM, and whether executive load is important in reducing this interference. It is possible that, if early sequence items require focused attention for active maintenance, its withdrawal through concurrent task manipulation increases the likelihood that distractor stimuli will then interfere.

### Method

#### Participants

Twenty-four undergraduate students (15 females) aged between 19 and 35 years took part, for financial payment or course credit. The study was approved by the research ethics committee at the Department of Psychology, University of York.

#### Materials, design, and procedure

Overall design and materials were drawn from Experiments 1, 3, and 5. Each condition started with 4 practice trials, followed by 30 test trials (15 no-distractor and 15 distractor). Probed serial position was pseudorandomized, with the constraint that each of the serial positions was assessed an equal number of times in each block (i.e., five times in each of the no-distractor and distractor trials, in each block).

**Figure 10 fig10:**
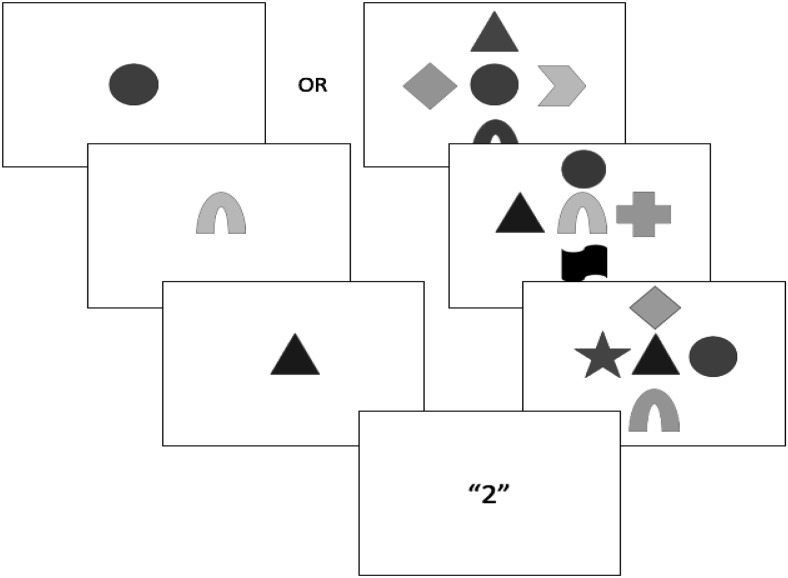
Schematic illustration of presentation and test method in Experiment 6.

Trial procedure is illustrated in Figure 10. Each of the three target stimuli was serially presented at screen center for 1,000 ms, with interstimulus intervals of 250 ms. In ‘distractor’ trials, each target stimulus was surrounded by four additional to-be-ignored stimuli, presented above, below, and to the left and right of the central target stimulus, each at a distance of approximately 0.45° visual angle. The three target (and distracter) displays were followed by a blank screen retention interval of 1,000 ms. An auditory recall probe was then played through the computer speakers. This consisted of the number “1,” “2,” or “3,” voiced by a female native English speaker. The participant was required to verbally recall the target stimulus that appeared in the serial position corresponding to the recall probe digit; thus, if the recall probe was “1,” recall of the first target in the sequence was required.[Fig-anchor fig10]

### Results and Discussion

**Figure 11 fig11:**
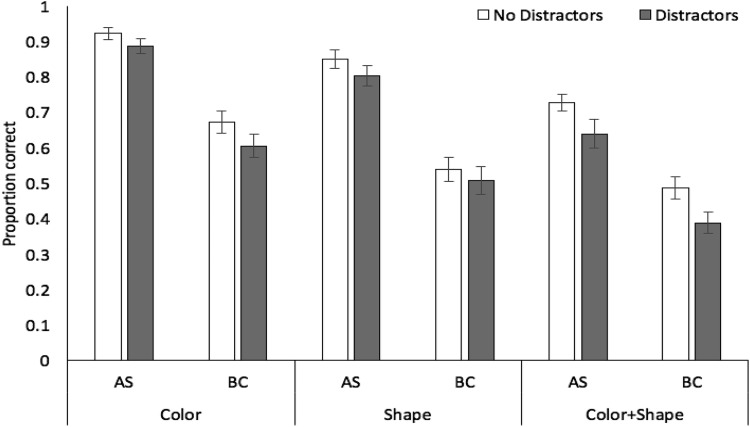
Proportion correct (with standard error in error bars) in Experiment 6 as a function of stimulus condition, distractors, and concurrent task.

Proportion correct in each condition is displayed in Figure 11. A 3 × 2 × 2 analysis of variance revealed significant effects of stimulus condition, *F*(2, 46) = 59.35, *MSE* = .02, *p* < .001, η_*p*_^2^ = .72, with recall accuracy higher for color (mean .77, *SE* .03) than shape (.78, .03) or color + shape (.56, .03), and the latter two conditions also differing (*p* < .001 in all cases, Bonferroni-Holm corrected). There were also significant effects of concurrent task, *F*(1, 23) = 117.96, *MSE* = .05, *p* < .001, η_*p*_^2^ = .84, and distractors, *F*(1, 23) = 16.90, *MSE* = .02, *p* < .001, η_*p*_^2^ = .42. The interaction between concurrent task and distractors was not significant, *F*(1, 23) = .11, *MSE* = .01, *p* = .74, η_*p*_^2^ = .01. Similarly, the interactions between stimulus condition and distractors, *F*(2, 46) = 1.66, *MSE* = .01, *p* = .20, η_*p*_^2^ = .07, stimulus condition and concurrent task, *F*(2, 46) = 1.42, *MSE* = .01, *p* = .25, η_*p*_^2^ = .06, were not significant, nor was the three-way interaction *F*(2, 46) = .34, *MSE* = .01, *p* = .71, η_*p*_^2^ = .02. Bayesian ANOVA indicated strongest support for the model just containing the main effects (BF >1,000 vs. the null-only model), preferring this over the model with main effects plus the distractor by task interaction by a factor of 5.2:1.[Fig-anchor fig11]

**Figure 12 fig12:**
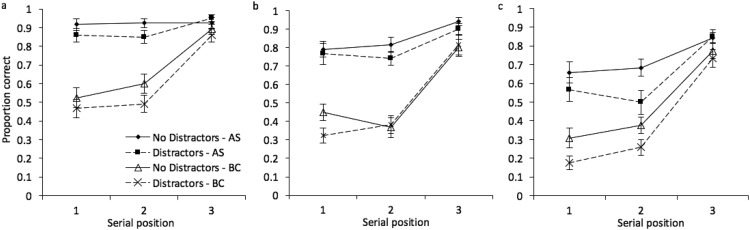
Proportion correct (with standard error in error bars) at each serial position in Experiment 6, for (a) color, (b) shape, and (c) color + shape conditions.

It is also of interest to examine performance by serial position, to explore whether the impact of each experimental factor, and the relationship between these factors, changes across the sequence. Mean proportion correct at each serial position is illustrated in Figure 12. A 3 × 2 × 2 × 3 repeated measures ANOVA revealed, in addition to the outcomes already described in the above analysis, a significant effect of serial position, *F*(2, 46) = 132.10, *MSE* = .03, *p* < .001, η_*p*_^2^ = .85. Serial position also significantly interacted with stimulus condition, *F*(4, 92) = 5.17, *MSE* = .04, *p* = .001, η_*p*_^2^ = .18, with concurrent task, *F*(2, 46) = 28.12, *MSE* = .03, *p* < .001, η_*p*_^2^ = .55, and with distractors, *F*(2, 46) = 4.89, *MSE* = .02, *p* = .012, η_*p*_^2^ = .18. There were no additional significant interactions (*p* > .15 in all cases). A Bayesian ANOVA preferred the model that included all main effects, plus interactions between stimulus condition and serial position, and concurrent task and serial position (BF >1,000 vs. the null-only model). There was no strong support for the inclusion of two-, three-, or four-way interactions between distractors and any other factor.[Fig-anchor fig12]

Backward counting performance (see Table 1) was also examined. A 3 × 2 repeated measures ANOVA revealed no effects of stimulus condition, *F*(2, 46) = 1.26, *MSE* = .11, *p* = .29, η_*p*_^2^ = .05, distractors, *F*(1, 23) = .80, *MSE* = .06, *p* = .38, η_*p*_^2^ = .03, or the interaction, *F*(2, 46) = .46, *MSE* = .05, *p* = .63, η_*p*_^2^ = .02. A Bayesian ANOVA indicated strongest support for the null model, preferring this over the model also containing distractors by a factor of 4.3 to 1.

This experiment therefore replicated the general patterns of data observed from Experiments 1–5, using serial target presentation. Even when targets were presented one at a time, with earlier items needing to be retained while later ones are presented, independent impacts of executive load and distraction were observed. Furthermore, this outcome emerged for earlier and final sequence items. We would also note that this experiment successfully replicated other recently published findings, using a novel test procedure; we observed a final-item recency effect that is relatively invulnerable to executive load, while earlier items were more affected by this manipulation (Allen et al., 2014; Hu et al., 2016). In addition, if the color + shape condition is characterized as a task that potentially taps WM binding, we replicated our previous finding that binding is relatively more fragile and prone to loss from earlier sequence positions (Allen et al., 2006, 2014).

## Experiment 7

The final experiment was closely based on Experiment 6, with one change. Rather than always presenting targets and distractors in the same central location, items shifted in their locations between trials. This was implemented to examine whether outcomes replicate when participants are not able to simply focus on the single target location at screen center (as in Experiment 6), and instead have to shift attention to different points on the screen on each trial.

### Method

#### Participants

Twenty-four undergraduate students (17 females) aged between 19 and 29 years took part, for financial payment or course credit. The study was approved by the research ethics committee at the Department of Psychology, University of York.

#### Materials, design, and procedure

Methodology was closely based on Experiment 6, with the adjustment that the entire display configuration (target and distractors) shifted in its locations around the screen in an unpredictable manner between (but not within) trials. Thus, the central target and surrounding distractors always retained this relative configuration, but the whole set moved randomly to a new location for each trial, by a horizontal distance up to a maximum of 7.41° (from screen center) and a vertical distance up to 2.98°. This move was initiated for the first item in each sequence, with subsequent items in the sequence appearing in the same locations.

### Results and Discussion

**Figure 13 fig13:**
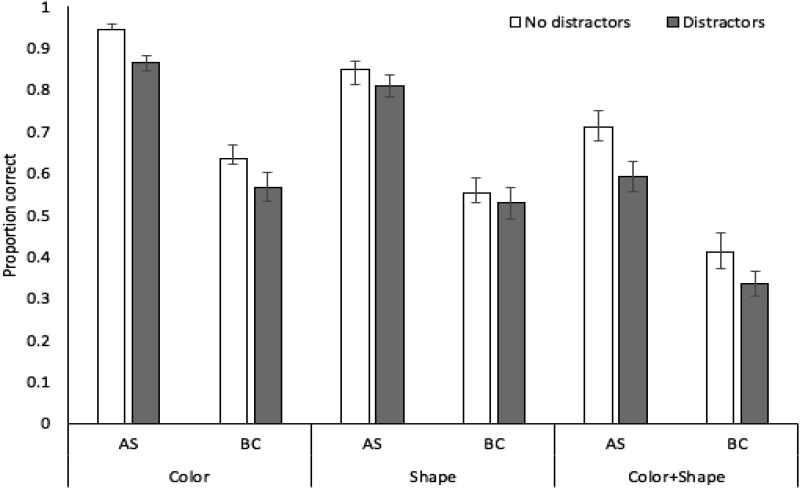
Proportion correct (with standard error in error bars) in Experiment 7 as a function of stimulus condition, distractors, and concurrent task.

Proportion correct in each condition is displayed in Figure 13. A 3 × 2 × 2 analysis of variance revealed significant effects of stimulus condition, *F*(2, 46) = 60.61, *MSE* = .02, *p* < .001, η_*p*_^2^ = .73, with recall accuracy higher for color (mean .75, *SE* .02) than shape (.68, .03) or color + shape (.51, .03), and the latter two conditions also differing (*p* ≤ .001 in all cases, Bonferroni-Holm corrected). There were also significant effects of concurrent task, *F*(1, 23) = 149.39, *MSE* = .05, *p* < .001, η_*p*_^2^ = .87, and distractors, *F*(1, 23) = 31.83, *MSE* = .02, *p* < .001, η_*p*_^2^ = .58. The interaction between concurrent task and distractors was not significant, *F*(1, 23) = 1.12, *MSE* = .01, *p* = .30, η_*p*_^2^ = .05. Similarly, the interactions between stimulus condition and distractors, *F*(2, 46) = 2.19, *MSE* = .01, *p* = .12, η_*p*_^2^ = .09, stimulus condition and concurrent task, *F*(2, 46) = .44, *MSE* = .01, *p* = .65, η_*p*_^2^ = .02, were not significant, nor was the three-way interaction *F*(2, 46) = .21, *MSE* = .01, *p* = .81, η_*p*_^2^ = .01. Bayesian ANOVA indicated strongest support for the model containing just the main effects (BF >1,000 vs. the null-only model), preferring this over the model with main effects plus the distractor by task interaction by a factor of 4.7:1.[Fig-anchor fig13]

**Figure 14 fig14:**
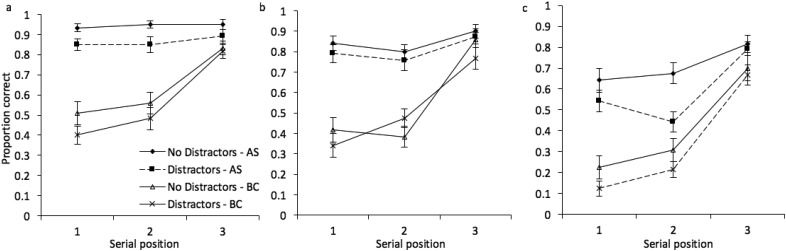
Proportion correct (with standard error in error bars) at each serial position in Experiment 7, for (a) color, (b) shape, and (c) color + shape conditions.

Proportion correct by serial position is illustrated in Figure 14. A 3 × 2 × 2 × 3 repeated measures ANOVA revealed, in addition to the outcomes already described above, a significant effect of serial position, *F*(2, 46) = 119.17, *MSE* = .05, *p* < .001, η_*p*_^2^ = .84. Serial position also significantly interacted with stimulus condition, *F*(4, 92) = 6.69, *MSE* = .04, *p* < .001, η_*p*_^2^ = .22, and with concurrent task, *F*(2, 46) = 47.73, *MSE* = .02, *p* < .001, η_*p*_^2^ = .68, but not with distractors, *F*(2, 46) = 1.63, *MSE* = .02, *p* = .21, η_*p*_^2^ = .07. There was a significant interaction between stimulus condition, distractors, and serial position, *F*(2, 46) = 2.63, *MSE* = .03, *p* = .04, η_*p*_^2^ = .10, but no other interactions reached the *p* < .05 cut off. A Bayesian ANOVA preferred the model that included all main effects, plus interactions between stimulus condition and serial position, and concurrent task and serial position (BF >1,000 vs. the null-only model). There was again no strong support for the inclusion of two-, three-, or four-way interactions between distractors and any other factor. Outcomes from Experiment 7 therefore closely replicate those observed in the preceding experiment.[Fig-anchor fig14]

Backward counting performance (see Table 1) was also examined. A 3 × 2 repeated measures ANOVA revealed no effects of stimulus condition, *F*(2, 46) = 07, *MSE* = .07, *p* = .93, η_*p*_^2^ = .01, distractors, *F*(1, 23) = .58, *MSE* = .02, *p* = .45, η_*p*_^2^ = .03, or the interaction, *F*(2, 46) = .02, *MSE* = .02, *p* = .98, η_*p*_^2^ = .01. A Bayesian ANOVA indicated strongest support for the null model, preferring this over the model also containing distractors by a factor of 4.9 to 1.

## General Discussion

Across seven experiments, using a range of stimuli, presentation formats, and timings, we observed highly reliable disruptive impacts of concurrent executive task and visual distraction. While impacts of verbal attentional load (e.g., Allen et al., 2006, 2012; Morey & Cowan, 2004) and visual distraction (e.g., McNab & Dolan, 2014) have previously been observed, this is the first study to demonstrate these effects within the same visual working memory paradigm. These findings demonstrate how working memory operates at the interface between perceptual selective attention that is externally oriented to information in the visual environment, and internally motivated attentional control (e.g., Chun et al., 2011; Yantis, 2000; Tamber-Rosenau, Esterman, Chiu, & Yantis, 2011). However, we found no clear or consistent evidence of an interactive relationship between these factors. Five of the seven experiments produced Bayes Factors providing at least moderate support for the absence of an interaction, while the remaining two experiments were equivocal regarding which model they preferred. To further confirm this overall pattern, and acknowledging the variation in method across experiments, proportion correct data from experiments sharing the same overall 3 × 2 × 2 design (i.e., all experiments except Experiment 4) were entered into frequentist and Bayesian ANOVA. These analyses produced a nonsignificant task by distractor interaction (*p* = .30, η_*p*_^2^ = .01), whereas the Bayes Factor analysis favored the main effects-only model over the model including the concurrent task by distractor interaction by a factor of 9.4 to 1. Similarly, comparing the 0- and 4-distractor trials for the color + shape condition in all seven experiments also produced a nonsignificant task by distractor interaction (*p* = .91, η_*p*_^2^ = .00), and a Bayes Factor favoring the main effects-only model by 9.3 to 1. Furthermore, while our primary experimental focus was on visual working memory performance, we also recorded backward counting scores as an additional measure. A Bayesian ANOVA of counting performance in these same experiments favored the null model over the model including the effect of distraction, by a factor of 13.5 to 1. Overall then, the weight of evidence from this experimental series indicates that withdrawal of executive control and imposition of visual distraction independently impacted on visual WM function.

These findings using encoding-based distraction are in line with recent work (Hu et al., 2016) suggesting that interference caused by a distractor suffix encountered during visual working memory retention (e.g., Allen et al., 2015; Brown, Niven, Logie, Rhodes, & Allen, 2017; Hu, Hitch Baddeley, Zhang, & Allen, 2014; Ueno, Allen, Baddeley, Hitch, & Saito, 2011) is not increased by the same type of concurrent attentional load implemented in the current studies. Specifically, Hu et al. (2016) found that, when targets were serially presented, a postsequence to-be-ignored suffix interfered with recall of the final target item, plus whichever item participants had been instructed to ‘prioritize.’ However, concurrent executive load did not mediate the magnitude of the suffix interference effect. In contrast, it did disrupt participants’ ability to selectively prioritize one target item above other targets. Thus, interference encountered either simultaneously with targets (the current experiments) or following target offset (Hu et al., 2016) is not critically influenced by executive attentional control, but selectively focusing on targets is. The observation of largely independent distractor processing and executive control effects would fit with the view that attention is not unitary, and instead should be considered a property of multiple perceptual and cognitive operations (Chun et al., 2011).

Outcomes from the present study are also in line with a general distinction between different forms of attentional control as set out in the load theory of selective attention proposed by Lavie and colleagues (e.g., de Fockert et al., 2001; Konstantinou et al., 2014; Lavie et al., 2004; Lavie, 1995, 2005, 2010). However, based on consistent evidence that concurrent executive/working memory load increases distractor interference in response competition tasks, Lavie has argued that working memory and executive control does play a role in minimizing distraction. The weight of evidence from the current study would run counter to this principle, and suggest that control of distraction may not operate in the same way across tasks measuring visual selective attention and working memory.

This study also informs research showing links between the ability to control and minimize visual distractor interference and working memory capacity. In general terms, these studies illustrate how working memory lies at the interface between voluntary and involuntary modes of attention (e.g., Fukuda & Vogel, 2009), and the present observation of disruption caused by both concurrent visual distraction and verbal attentional load are in accordance with this. Recent work has attempted to specify more precisely how visual working memory capacity might relate to performance in these kinds of measures. For example, Emrich and Busseri (2015) reanalyzed outcomes from McNab and Klingberg (2008) and Liesefeld et al. (2014), and found that individual differences in working memory capacity were predicted by filtering-related activity, rather than unnecessary storage of distractor items. Emrich and Busseri (2015) suggested that this filtering activity might reflect general top-down attentional control, and would be critical for the selection and processing of target items, even when to-be-ignored distractors are absent. Taking a slightly different approach, Shipstead et al. (2014) distinguished between memory for visual arrays with and without additional distraction. They found that these factors independently predicted variability in attentional control, and that only visual target memory, and not visual memory under distraction, correlated with working memory capacity. Together, these studies suggest that the processing of target information draws on general attentional control mechanisms, and is closely related to working memory capacity. This might fit with the finding from Hu et al. (2016) that selectively encoding and maintaining targets requires attention, and more broadly with the claims of Cowan (2001) that the focus of attention may operate as an active holding device in working memory.

Within the context of the present study, backward counting is a task that clearly draws on general top-down control, and is involved in focusing on targets within visual WM regardless of whether distractors are present. The disruptive effects observed in this and other studies (e.g., Allen et al., 2006, 2012) would therefore capture the same forms of general attentional mechanisms that are reflected in the possible relationship between visual working memory and broader working memory capacity, via the ability to encode, maintain, and retrieve goal-relevant targets. This form of attentional control appears to be relatively distinct from that involved in visual distractor suppression, and the unnecessary storage or otherwise of distractors may not be a critical element in this relationship. Instead, this may relate to attentional control in a way that is independent of target processing., perhaps reflecting domain-specific visuospatial attention.

Although the details of how distractors are controlled when concurrent with targets remain to be explored, one possibility is that a spatially oriented attentional spotlight or ‘zoom’ function operates during memory encoding. This type of model has been proposed in the context of spatial selective attention (Eriksen & Yeh, 1985; Eriksen & St. James, 1986; Kahneman, 1973; Posner & Peterson, 1990), and may operate during memory encoding in enabling the individual to focus on targets while filtering out nearby visual distraction. This would be required in any situation where a target is encountered in the presence of distraction, regardless of how many distractors were present (as was observed in Experiment 4). Furthermore, possibly in line with the unpredictably mixed target and distractor locations used in Experiment 3, it has been suggested that this attentional spotlight can be divided between noncontiguous spatial locations (Awh & Pashler, 2000; Müller, Malinowski, Gruber, & Hillyard, 2003). We acknowledge that the extent to which such studies relate to working memory tasks (featuring longer encoding and retention durations) requires further exploration. Nevertheless, the current findings suggest that if this type of spatially oriented selective attentional processes indeed operates within a visual working memory context, it is largely independent of executive control.

This general conclusion might appear to run counter to the claims of Engle and colleagues (e.g., Engle, Tuholski, Laughlin, & Conway, 1999; Kane & Engle, 2003), who have suggested that the control and inhibition of interference is a key feature of working memory function, and that attentional control mechanisms analogous to the central executive are central to this ability. The present findings would indicate that this might not apply to all forms of interference control or executive-based attention. Domain-general executive control functions loaded on by our backward counting task are clearly highly important for visual WM, as evidenced by the substantial and reliable effects on performance found in the seven experiments reported here, and in previous work (e.g., Allen et al., 2006, 2012, 2014; Brown & Brockmole, 2010; Hu et al., 2016), but appear to be less critical for preventing visual distractor interference. Given the proposed diversity of executive function (e.g., Miyake et al., 2000), and the suggestion to retire the concept of a monolithic ‘central executive’ (Logie, 2016), it is possible that while the form of executive control tapped by backward counting is not crucial for distractor control, tasks loading directly on other subcomponents of executive function may produce effects that are more consistently interactive with, rather than independent of, distraction interference. For example, using an individual differences approach, Friedman and Miyake (2004) have observed a close relationship between performance on response inhibition and distractor control tasks. One potentially fruitful avenue for future experimental research might lie in examining whether other concurrent load manipulations, designed to explicitly load on specific executive functions, are more successful in consistently reducing the ability to control distraction.

However, the aim in this experimental series was to examine how executive-based modality-general attentional control, rather than any more specialized cognitive function, might be involved in mediating the impacts of visual distraction. Furthermore, it is worth noting that executive function is characterized as demonstrating a unity as well as a diversity, with a high degree of relatedness between performance on measures of, for example, updating, task switching, inhibition, and dual-tasking (Miyake et al., 2000). If there are important commonalities between such subcomponents, and distractor control is indeed related to executive function, any executive load task should then affect this ability. On a broader note, few tasks if any are process-pure. We assume that concurrent backward counting draws on modality-general executive control and interferes with a range of processes associated with visual working memory performance. Given this, and the substantial impacts of concurrent counting on target memory performance that were consistently observed across the current experimental series, it is perhaps striking that it does not appear to consistently cause substantial and reliable interference to distractor control.

Finally, although the current study was primarily focused on attentional control manipulations, these were applied (in six of the seven experiments) across conditions requiring memory for color, shape, and color + shape combinations. Comparison of the two single feature tasks with the condition requiring recall of both dimensions for a correct response yields a possible examination of feature binding ability (Gajewski & Brockmole, 2006). In this light, we found no clear evidence of increased concurrent task or distraction effects on binding/object memory versus memory for single features across the six experiments that involved the relevant conditions. This extends previous observations of equivalent executive load effects on feature and binding memory (Allen et al., 2006, 2012, 2014) to a different response task, to the use of meaningful stimuli (Experiment 2) and to a delay-based load manipulation (Experiment 5, see also Morey & Bieler, 2013). It also indicates that multifeature object binding memory is not more vulnerable to concurrent distraction than memory for single features.

Overall then, examining memory for colors, shapes, and colored shape combinations, and across a range of experiments, we find consistent evidence that concurrent executive and perceptual load manipulations interfere with task performance, indicating both domain-general executive control and visual selective attention to be key to visual working memory. Furthermore, the weight of evidence supports the conclusion that these forms of attentional control operate in a relatively independent manner, with executive control not a critical factor in the ability to reduce interference caused by visual distractor stimuli. Future work should aim to explore the factors underlying the previously observed relationship between working memory capacity, executive function, selective attention, and control of distraction.

## Figures and Tables

**Table 1 tbl1:** Mean Number of Counting Responses (and Standard Error) in Each Condition Across Experiments 1–7

Experiment	Color	Shape	Color + Shape
Experiment 1			
No distractors	2.69 (.02)	2.62 (.02)	2.61 (.02)
Distractors	2.65 (.02)	2.57 (.02)	2.54 (.02)
Experiment 2			
No distractors	2.26 (.11)	2.22 (.11)	2.34 (.12)
Distractors	2.23 (.10)	2.22 (.12)	2.36 (.12)
Experiment 3			
No distractors	2.79 (.10)	2.80 (.10)	2.83 (.08)
Distractors	2.79 (.10)	2.83 (.09)	2.67 (.19)
Experiment 4			
No distractors	—	—	2.54 (.08)
1 distractor	—	—	2.52 (.08)
4 distractors	—	—	2.51 (.09)
8 distractors	—	—	2.53 (.09)
Experiment 5			
No distractors	2.26 (.13)	2.25 (.15)	2.15 (.16)
Distractors	2.36 (.15)	2.23 (.13)	2.22 (.15)
Experiment 6			
No distractors	3.82 (.18)	3.90 (.19)	3.86 (.20)
Distractors	3.82 (.17)	3.93 (.19)	3.94 (.20)
Experiment 7			
No distractors	3.58 (.12)	3.58 (.11)	3.60 (.13)
Distractors	3.60 (.12)	3.60 (.12)	3.62 (.14)

## Footnotes

1 Analysis of transformed data sets from each of the subsequent experiments was also carried out. This analysis produced outcomes equivalent to those observed using raw proportion correct (with one minor exception, in Experiment 3).
